# Bioinformatic analysis of microRNA and mRNA Regulation in peripheral blood mononuclear cells of patients with chronic obstructive pulmonary disease

**DOI:** 10.1186/s12931-016-0486-5

**Published:** 2017-01-05

**Authors:** Xiaomin Dang, Xiaoyan Qu, Weijia Wang, Chongbing Liao, Ying Li, Xiaojin Zhang, Dan Xu, Carolyn J. Baglole, Dong Shang, Ying Chang

**Affiliations:** 1 Center for Translational Medicine, The Key Laboratory of Biomedical Information Engineering of Ministry of Education, School of Life Science and Technology and Frontier Institute of Science and Technology, Xi’an Jiaotong University, Xi’an, 710049 Shaanxi province China; 2 Department of Respiration, The First Affiliated Hospital, Xi’an Jiaotong University, Xi’an, 710061 Shaanxi province China; 3 Meakins-Christie Laboratories and Respiratory Division, Health Centre and Department of Medicine, McGill University, Montreal, QC Canada

**Keywords:** miRNA, COPD, PBMC, Microarray, MicroRNA

## Abstract

**Background:**

Chronic obstructive pulmonary disease (COPD) is a progressive, irreversible chronic inflammatory disorder typified by increased recruitment of monocytes, lymphocytes and neutrophils. Because of this, as well as the convenience of peripheral blood nuclear cells (PBMCs) assessments, miRNA profiling of PBMCs has drawn increasing attention in recent years for various disease. Therefore, we analyzed miRNA and mRNA profiles to understand their regulatory network between COPD subjects versus smokers without airflow limitation.

**Methods:**

miRNA and mRNA profiling of PBMCs from pooled 17 smokers and 14 COPD subjects was detected by high-throughput microarray. The expression of dysregulated miRNAs were validated by q-PCR. The miRNA targets in dysregulated mRNAs were predicted and the pathway enrichment was analyzed.

**Results:**

miRNA microarray showed that 8 miRNAs were up-regulated and 3 miRNAs were down-regulated in COPD subjects compared with smokers; the upregulation of miR-24-3p, miR-93-5p, miR-320a and miR-320b and the downregulation of miR-1273 g-3p were then validated. Bioinformatic analysis of regulatory network between miRNA and mRNA showed that NOD and TLR were the most enriched pathways. miR-24-3p was predicted to regulate IL-18, IL-1β, TNF, CCL3 and CCL4 and miR-93-5p to regulate IκBα.

**Conclusions:**

The expression of miRNA and mRNA were dysregulated in PBMCs of COPD patients compared with smokers without airflow limitation. The regulation network between the dysregulated miRNA and mRNA may provide potential therapeutic targets for COPD.

## Background

Chronic obstructive pulmonary disease (COPD) is a progressive, irreversible chronic inflammatory disorder. Caused predominantly by cigarette smoking, COPD is one of the leading causes of mortality globally [1]. Although cigarette smoking is the major cause of COPD, there is currently no satisfactory therapy to treat individuals once the disease is established. Inflammation plays a pivotal role in the disease process, with CD8^+^ T lymphocytes, neutrophils and macrophages being the main type of immune cells of local inflammatory milieu of COPD [2]. Different immunoregulatory properties of T cells and monocytes have been demonstrated in COPD patients [3], and the inflammatory response in the lungs of COPD patients is strongly linked to tissue destruction and alveolar airspace enlargement [4], in part due to the loss of lung structural cells due to heightened apoptosis.

microRNAs (miRNAs) are a growing family of small non-coding RNAs (approximately 19 to 25 nucleotides long) with a regulatory function on gene expression [5]. Through binding to the 3′ untranslated region (3′ UTR) of target messenger RNAs, miRNAs can lead to direct inhibition of protein translation or degradation of messenger RNA [5]. In addition, miRNAs can alter gene expression by targeting transcription factors and DNA methyltransferases. In this way, miRNAs work as post-transcriptional regulators of gene expression and control various cellular processes such as differentiation, proliferation, and cell-cell interaction. The dysregulation of miRNAs is linked to a wide spectrum of diseases, including proliferative vascular disease, cardiac disorders, lung diseases, kidney diseases, diabetes mellitus, fibrosis and cancer [6–8].

A few studies have been conducted to reveal the dysregulation and role of miRNAs in COPD. Ezzie et al. [9] compared the miRNA expression in lung tissue derived from smokers with and without COPD and identified 70 differentially expressed miRNAs. A report by Pottelberge et al. [10] demonstrated that 34 miRNAs were differentially expressed between never-smokers and current smokers without airflow limitation, and 8 miRNAs were expressed at a significantly lower level in current-smoking patients with COPD compared with never-smokers without airflow limitation. Another study showed that microRNA-34c is associated with emphysema severity in COPD [11]. Sato et al. [12] observed reduced miR-146a expression in lung fibroblasts of patients with COPD and showed that miR-146a deficiency increased the expression of PGE_2_ through depression of the miR-146a target COX-2. Finally, Lewis et al. [13] found downregulated miR-1 expression in quadriceps muscles and speculated that this is linked to the muscle weakness observed in COPD.

On account of the convenience of peripheral blood nuclear cells (PBMC) assessments, the miRNA profiling of PBMC has drawn increasing attention in recent years in various diseases such as cancer [14, 15], Alzheimer’s Disease [16], diabetes [17] and autoimmune disease [18]. However, there is no report regarding miRNA expression profiles of PBMC in COPD patients. Therefore, in this study, we sought to determine if miRNAs were differentially expressed in PBMCs of COPD patients and if miRNA expression may be linked to dysregulated mRNA expression relevant to the pathogenesis of COPD. We analyzed miRNA and mRNA expression profiles in PBMCs from COPD patients versus smokers without airflow limitation. The mRNA targets of the dysregulated miRNA were predicted and pathway enrichment was analyzed. We identified a signature of COPD-associated miRNA, such that miR-24-3p, miR-93-5p, miR-320a, miR-320b and miR-1273g-3p were significantly dysregulated in COPD patients. Bioinformatic analysis between miRNA and predicted mRNA showed that NOD and TLR were the most enriched pathways. In NOD pathway, miR-24-3p was predicted to regulate IL18/IL1B/TNF, and miR-93-5p to regulate NLRP3/IL6/NFKBIA. In TLR pathway, miR-24-3p was predicted to regulate CCL3/CCL4/IL1B/TNF, and miR-93-5p to regulate IL6/CXCL10/NFKBIA.

## Methods

### Subjects

Peripheral venous blood was taken in heparin-coated tubes from 17 smokers without airflow limitation and 14 COPD subjects at Department of Respiration, The First Affiliated Hospital, Xi’an Jiaotong University, Xi’an, China. COPD subjects were eligible for this study if they met the following criteria: age ≥ 50 and ≤76 years; smoking history (≥20 pack-years); post-bronchodilator FEV_1_ ≥ 25% of predicted value and post-bronchodilator FEV_1_/forced vital capacity (FVC) ≤ 0.70; no history of asthma, atopy (as assessed by an allergy skin prick test during screening) or any other active lung disease. Patients on home oxygen or with raised carbon dioxide tension (>44 mmHg), α_1_-antitrypsin deficiency, recent exacerbation (in the last 4 weeks), an uncontrolled medical condition or hypersensitivity to inhaled corticosteroids and bronchodilators were not eligible for the study. All smokers without airflow limitation met the following criteria: age ≥ 42 and ≤75 years, Post-BD FEV1% predicted >80, no diagnostic cancer, diabetes, cardiovascular disease and hypertension, no use of inhaled or oral corticosteroids in the previous 6 months, no atopy, and no respiratory tract infection 1 month prior to the study. Patient characteristics are in Table 1. The experimental procedures were performed with ethical approval from the Research Ethics Boards of The First Affiliated Hospital, Xi’an Jiaotong University (2015–015).

**Table 1:** Clinical characteristics of smokers without airflow limitation and COPD patients

	Smokers	COPD
Number	17	14
Age	56 ± 17	69 ± 8
Male/Female	17/0	13/1
Current/ex-smokers	14/3	8/6
Pack-years	23 ± 3	27 ± 6
Post-BD FEV1% predicted	92.9 ± 9.0	29.0 ± 10.6
GOLD Stage		
I	-	0
II	-	2
III-IV	-	12

Data are presented as Means ± SD. *BD* bronchodilator, *FEV1* forced expiratory volume in 1 s

### PBMC isolation and RNA extraction

PBMCs were isolated from venous blood by density gradient centrifugation over Ficoll-Paque PLUS reagent (GE Healthcare, Uppsala, Sweden) and suspended in QIAzol Lysis Reagent (Qiagen, Dusseldorf, Germany). Total RNA was extracted using miRNeasy Mini Kit (Qiagen) according to the manufacturer’s procedure. RNA integrity was determined by formaldehyde denaturing gel electrophoresis.

### miRNA and gene expression microarray

Equal amount of RNA sample from each smokers (*N* = 17) and COPD patients (*N* = 14) was pooled respectively for miRNA profiling assay using Affymetrix GeneChip miRNA Array v.4.0 (Affymetrix, Santa Clara, CA, USA) by Capitalbiotech company, Beijing, China. Briefly, after labeled with Biotin, the total RNA was subsequently hybridized overnight. The GeneChip® miRNA 4.0 arrays, containing 30,424 total mature miRNA probe sets including 2,588 mature human miRNAs and miRNAs of 202 other organisms, were washed and stained using the Affymetrix GeneChip Hybridization Wash and Stain Kit and were then scanned with the Affymetrix GeneChip® Scanner 3000 (Affymetrix, Santa Clara, CA, USA).

### Messenger RNA microarray

Samples were prepared for mRNA microarray analysis using Agilent Human Gene Expression Microarray V4.0 (Santa Clara, California, USA). Hybridized slides were then washed and scanned with Agilent Microarray Scanner System (G2565CA).

### Data analysis

The miRNA and mRNA array data were analyzed for data summarization, normalization and quality control using GeneSpring V11.5 software (Agilent). To select differentially expressed genes, we used threshold values of >2 fold change. The data were Log2 transformed and median centred by genes using the Adjust Data function of CLUSTER 3.0 software. Further analysis was performed by hierarchical clustering with average linkages. Finally, we performed tree visualization using Java Treeview (Stanford University School of Medicine, Stanford, CA, USA).

### Quantitative reverse transcription PCR validation

Independent assays were performed using quantitative reverse transcription PCR (qRT-PCR) on all patient samples for individual miRNA (miR-24-3p, miR-93-5p, miR-320a, miR-320b, miR-191-5p, let-7b-5p, miR-342-3p, miR-92a-3p, miR-3613-3p, miR-1273 g-3p and miR-4668-5p) (Qiagen) and predicted target genes (IL18, IL1B, TNF, CCL3, CCL4, NLRP3, IL6, NFKBIA and CXCL10) (Bio-rad, Foster city, USA). In addition, the expression of miR-24-3p, miR-93-5p, miR-320a, miR-320b and miR-1273 g-3p was detected on the isolated different cell types including CD4^+^ T cells, CD8^+^ T cells, CD20^+^ B cells and CD14^+^ monocytes from PBMCs in some COPD patients by positive selection (Anti-PE MicroBeads UltraPure, Miltenyi Biotec, Teterow, Germany). Data were presented relative to U6 for miRNA and β-actin for target genes based on calculations of 2^(−σσCt)^. The primer sequences for target genes were listed in Table 2. Statistical significance was defined as *p* < 0.05 as measured by the *t* test using GraphPad Prism 5 software (GraphPad, San Diego, CA, USA).

**Table 2:** The sequence of primers for real-time PCR

Gene	Sequence (5′-3′)	Direction
IL18	GACCAAGGAAATCGGCCTCTA	Forward
	AGTTACAGCCATACCTCTAGGC	Reverse
IL1B	CTCTGGGATTCTCTTCAGCCA	Forward
	AATAAGCCATCATTTCACTGGCG	Reverse
TNF	GCTGCACTTTGGAGTGATCG	Forward
	GGCCAGAGGGCTGATTAGAG	Reverse
CCL3	GCTCTCTGCAACCAGTTCTCT	Forward
	GGCTGCTCGTCTCAAAGTAGT	Reverse
CCL4	CTCCCAGCCAGCTGTGGTATTC	Forward
	CCAGGATTCACTGGGATCAGC	Reverse
NLRP3	ATGAGAGTGTTGTGTGAAACGC	Forward
	GAGATGTCGAAGCAGCACTCA	Reverse
IL6	CAGACAGCCACTCACCTCTTC	Forward
	CAGGTTGTTTTCTGCCAGTGC	Reverse
NFKBIA	GTACGAGCAGATGGTCAAGGA	Forward
	GGTCAGTGCCTTTTCTTCATGG	Reverse
CXCL10	GCCATTCTGATTTGCTGCCTT	Forward
	ACTAATGCTGATGCAGGTACAG	Reverse
β-actin	TACCTCATGAAGATCCTCACC	Forward
	TTTCGTGGATGCCACAGGAC	Reverse

### Target prediction and network analysis

The target genes for miRNAs were predicted using miRanda, MirTarget2, PicTar, PITA and TargetScan. The regulation network diagram between miRNAs and mRNAs was generated using Cytoscape. Based on the data of mRNA array, the predicted target genes that negatively regulated by the validated dysregulated miRNAs were selected for the further pathway enrichment analysis. The DAVID [19] online analysis tool was used and the significant enrichment threshold was P value of Modified Fisher exact less than 0.05 and enriched gene count more than 2.

## Results

### MicroRNAs are dysregulated in PBMCs of COPD patients

**Fig. 1:**
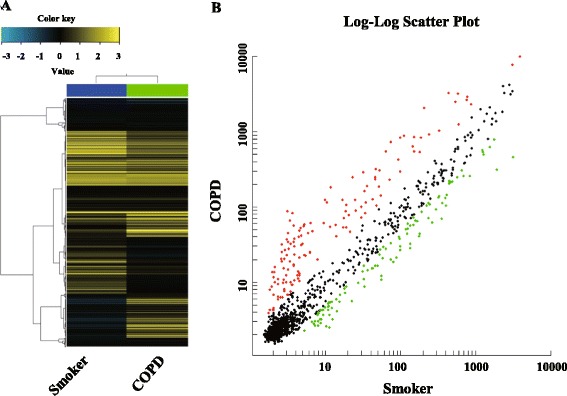
Hierarchical clustering and scatter plot result of differentially expressed miRNAs in PBMCs from smokers and COPD patients. **a** Hierarchical clustering image of miRNA expression of pooled RNA samples from PBMCs of COPD patients compared to smokers without airflow limitation. **b** Scatter plot of miRNA expression of PBMCs of COPD patients compared to smokers without airflow limitation. Red and green colored dots represent up- and down- regulated miRNAs in scatter plot, respectively

We investigated the miRNA profiling in pooled PBMCs from 17 smokers without airflow limitation and 14 COPD patients (Fig. 1). Compared with smokers, there were 103 up-regulated and 34 down-regulated miRNAs in COPD patients. We selected the dysregulated miRNAs with differences in the fluorescence intensity higher than 1000 between the smokers and COPD patients. As shown in Table 3, there were a total of 8 up-regulated and 3 down-regulated miRNA; these were selected for the further validation and analysis.

**Table 3:** Selected dysregulated miRNAs in COPD patients compared with smokers without COPD

Upregulation		Downregulation	
miRNA	Fold change	miRNA	Fold change
hsa-miR-24-3p	9.80	hsa-miR-3613-3p	0.14
hsa-miR-93-5p	9.73	hsa-miR-1273 g-3p	0.16
hsa-miR-320a	5.52	hsa-miR-4668-5p	0.38
hsa-miR-320b	4.62		
hsa-miR-191-5p	3.73		
hsa-let-7b-5p	3.43		
hsa-miR-342-3p	2.62		
hsa-miR-92a-3p	2.06		

The miRNAs with difference of fluorescence intensity higher than 1000 was selected

### Validation of dysregulated miRNAs in PBMCs of COPD patients

**Fig. 2:**
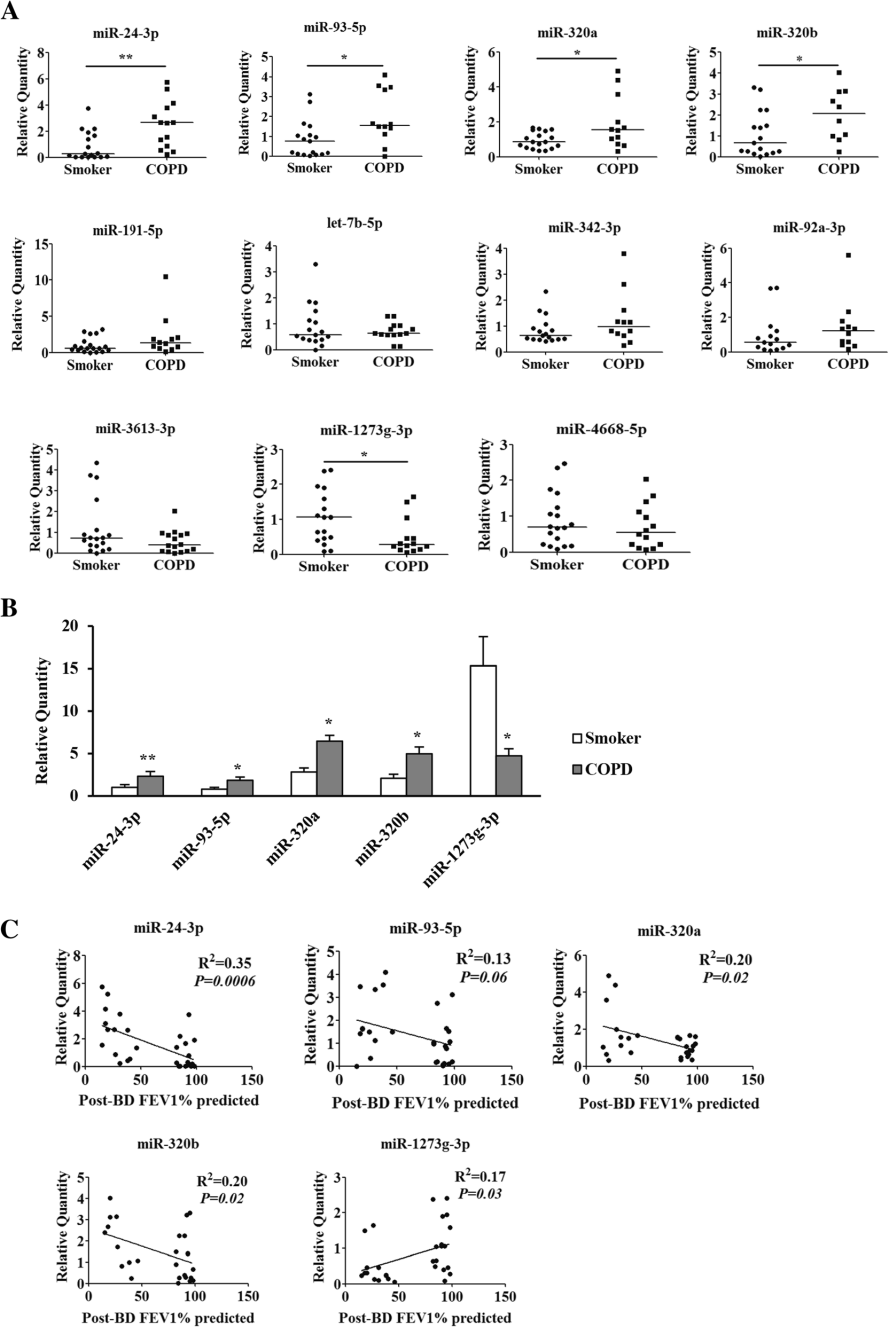
Validation of differentially expressed miRNAs. **a** Expression of selected miRNAs in PBMCs of smokers and COPD patients. qRT-PCR was performed on the same RNA samples (17smokers and 14 COPD patients) as microarray analysis. Data are presented as 2^(−σσCt)^ relative to U6. ^*^ *P* < 0.05, ^**^ *P* < 0.01 compared with smokers by Mann Whitney *U* test. **b** Relative abundance of differentially expressed miRNAs in PBMCs of smokers and COPD patients. ^*^ *P* < 0.05, ^**^ *P* < 0.01 compared with smokers. **c** Correlation analysis between miRNA expression and FEV1% predicted

Among the selected 11 dysregulated miRNAs in COPD patients versus smokers, we validated the expression of 5 miRNAs (miR-24-3p, miR-93-5p, miR-320a, miR-320b and miR-1273 g-3p) in smokers and COPD patients by qRT-PCR, as shown in Fig. 2a. MiR-1273 g-3p was the most highly expressed miRNA in PBMCs of smokers, while miR-320a and miR-320b had the higher relative abundance in PBMCs of COPD patients (Fig. 2b). The correlation analysis between miRNA expression and FEV1% predicted showed that significant negative relevance appeared in the expression of miR-24-3p, miR-320a and miR-320b and positive relevance appeared in miR-1273 g-3p (Fig. 2c). However, there is no difference in miRNA sets between ex- and current smokers in the smokers and COPD groups.

### Verification of expression of dysregulated miRNAs in different cell types of PBMCs

**Fig. 3:**
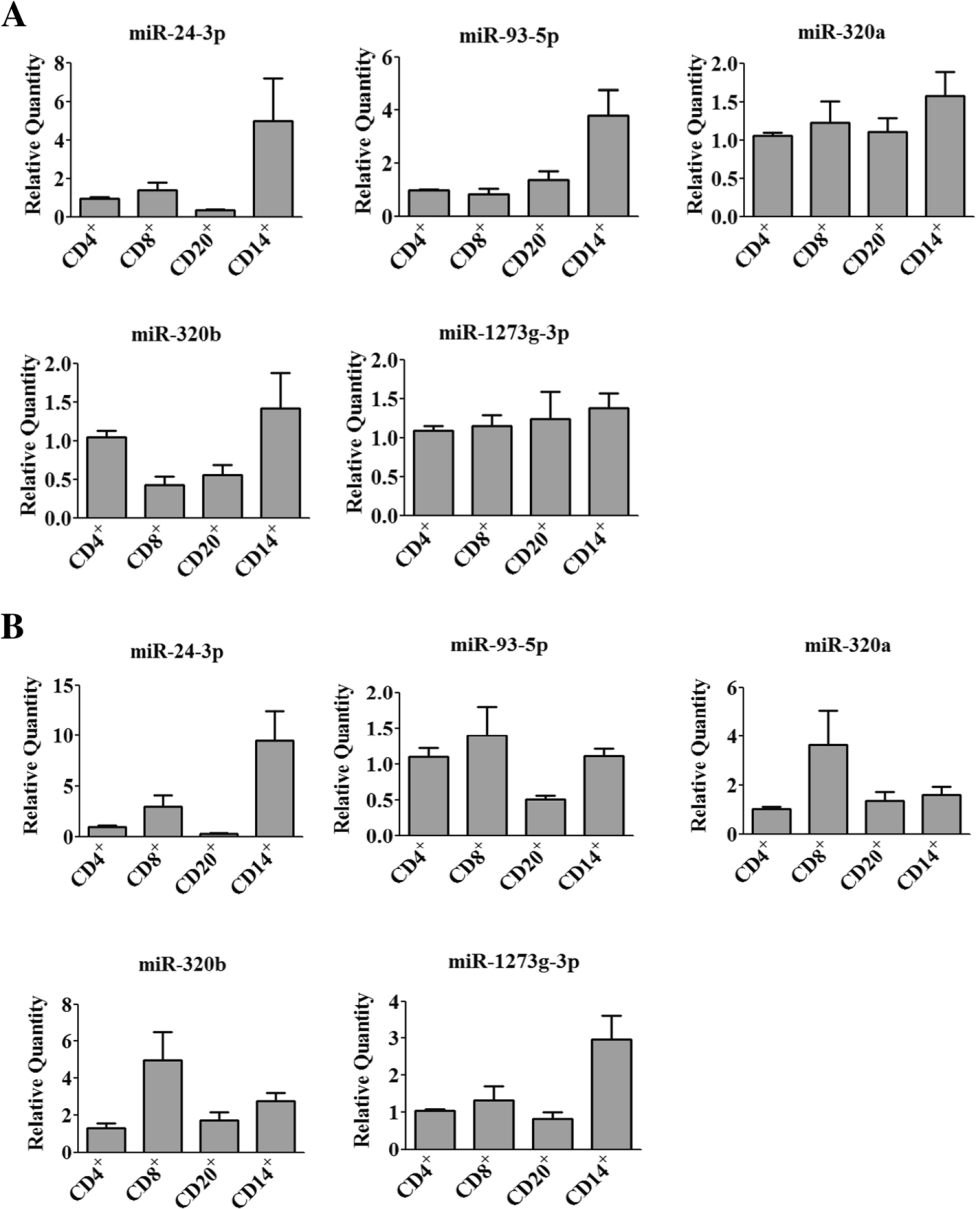
Expression of miRNAs in the isolated different cell types of PBMCs from smokers (**a**) and COPD patients (**b**). The expression of miR-24-3p, miR-93-5p, miR-320a, miR-320b and miR-1273g-3p was examined by qRT-PCR on CD4^+^ T lymphocytes, CD8^+^ T lymphocytes, CD20^+^ B lymphocytes and CD14^+^ monocytes from smokers and COPD patients. Data are presented as 2^(−σσCt)^ relative to β-actin

PBMCs consist mainly of T lymphocytes, B lymphocytes and monocytes. We therefore analyzed the expression of dysregulated miRNAs in the isolated different cell types including CD4^+^ T cells, CD8^+^ T cells, CD20^+^ B cells and CD14^+^ monocytes from PBMCs of smokers and COPD patients. MiR-24-3p was consistently expressed higher in monocytes. In smokers, miR-93-5p was mainly expressed in monocytes, while it was equally expressed in CD4 ^+^ T cells, CD8 ^+^ T cells and monocytes in COPD patients. CD8 ^+^ T cells mainly contribute to the increased expression of miR-320a and miR-320b in COPD patients. In COPD patients, more miR-1273 g-3p was expressed in monocytes, suggesting that the expression was decreased in CD4 ^+^ T cells, CD8 ^+^ T cells and CD20 ^+^ B cells in COPD (Fig. 3).

### mRNAs are dysregulated in PBMCs of COPD patients

**Fig. 4:**
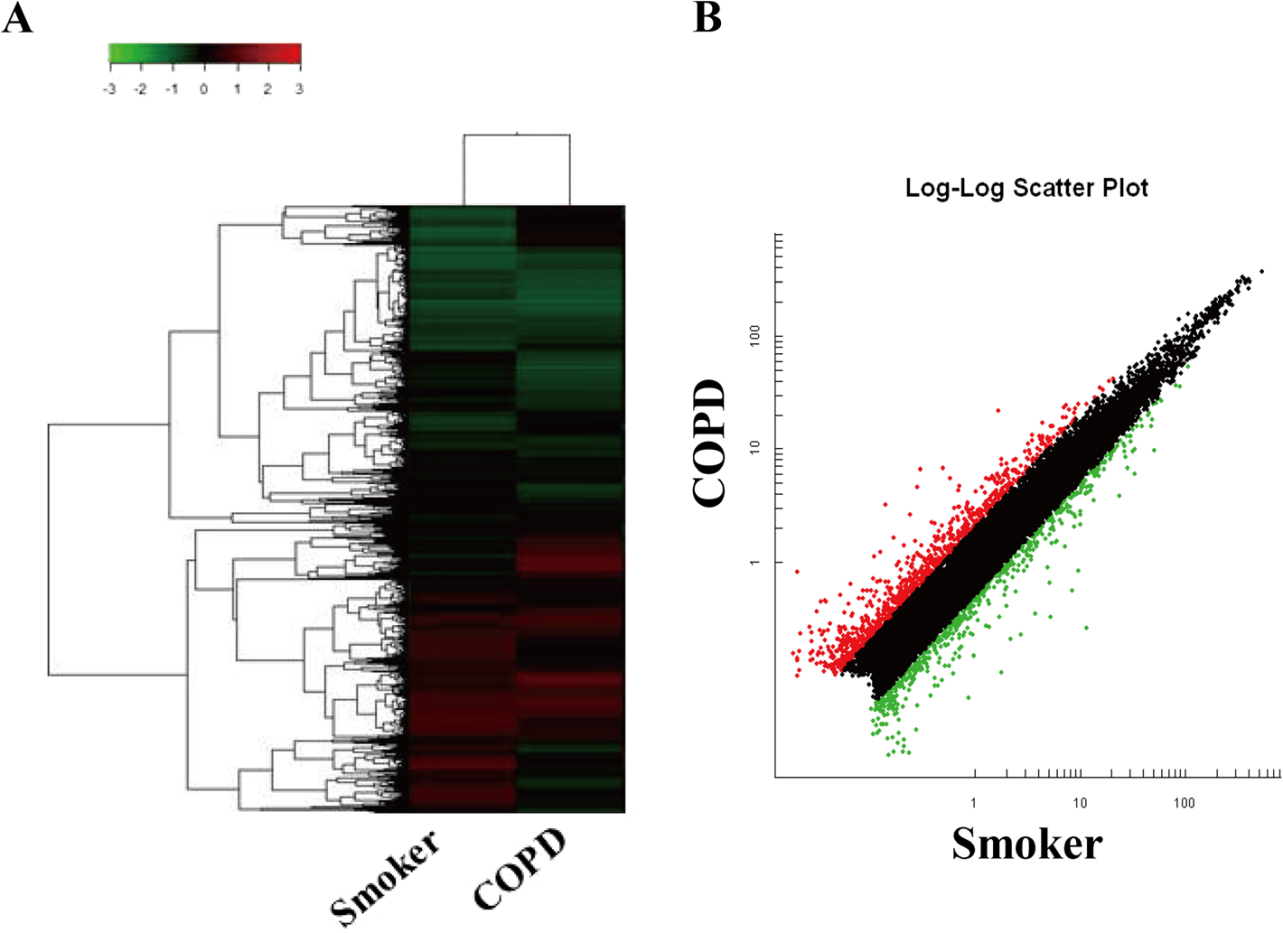
Hierarchical clustering and scatter plot result of differentially expressed mRNAs in PBMCs from smokers and COPD patients. **a** Hierarchical clustering image of mRNA expression of pooled RNA samples from PBMCs of COPD patients compared to smokers without airflow limitation. **b** Scatter plot of mRNA expression of PBMCs of COPD patients compared to smokers without airflow limitation. Red and green colored dots represent up- and down- regulated mRNAs in scatter plot, respectively

We performed a parallel mRNA microarray study to compare the mRNA expression between the smokers and COPD patients. The results revealed a total of 1508 genes that differed in expression between the two groups (fold change >2; Fig. 4), where 164 mRNAs were up-regulated and 137 were down-regulated when filtered by fluorescence intensity difference higher than 1000. Table 4 shows the 20 genes with the largest fold changes and their known biological functions.

**Table 4:** Top 10 dysregulated mRNAs in COPD patients compared with smokers without COPD

Gene symbol	Gene name	Fold change COPD/Smokers	Function
Upregulation			
CD177	CD177 molecule	22.59	Leukocyte migration
MUC17	Mucin 17, cell surface associated	21.54	Extracellular matrix constituent
IL1R2	Interleukin 1 receptor, type II	10.96	Decoy receptor, inhibits the activity of IL-1
SARDH	Sarcosine dehydrogenase	9.52	Mitochondrial matrix
EGR3	Early growth response 3	6.75	Positive regulation of endothelial cell proliferation
AREG	Amphiregulin	6.16	EGF family, promote the growth of normal epithelial cells
SLC6A2	Solute carrier family 6 (neurotransmitter transporter, noradrenalin), member 2	4.70	Sodium symporter
TMEM167A	Transmembrane protein 167A	4.55	Golgi apparatus
KCNJ15	Potassium inwardly-rectifying channel, subfamily J, member 15	4.42	Potassium channel activity
FCHO1	FCH domain only 1	4.39	Clathrin-mediated endocytosis
Downregulation			
IL1A	Interleukin 1, alpha	−44.69	Immune response
IL6	Interleukin 6 (interferon, beta 2)	−16.14	Pro-inflammatory and anti-inflammatory role
CXCL10	Chemokine (C-X-C motif) ligand 10	−15.08	leukocyte chemotaxis
TNF	Tumor necrosis factor	−11.79	Inflammation, cause apoptosis
CCL20	Chemokine (C-C motif) ligand 20	−7.94	Lymphocytes chemotaxis
CCL4	Chemokine (C-C motif) ligand 4	−6.19	Leukocyte chemotaxis
CCL3L3	Chemokine (C-C motif) ligand 3-like 3	−5.28	Leukocyte chemotaxis
C9orf7	Chromosome 9 open reading frame 7	−5.23	Calcium channel activity
IL1RN	Interleukin 1 receptor antagonist	−4.69	Inhibition of the activities of IL-1
RNF19B	Ring finger protein 19B	−4.17	Cytotoxic effects of natural killer (NK) cells

### Regulation network of dysregulated miRNAs and mRNAs

**Fig. 5:**
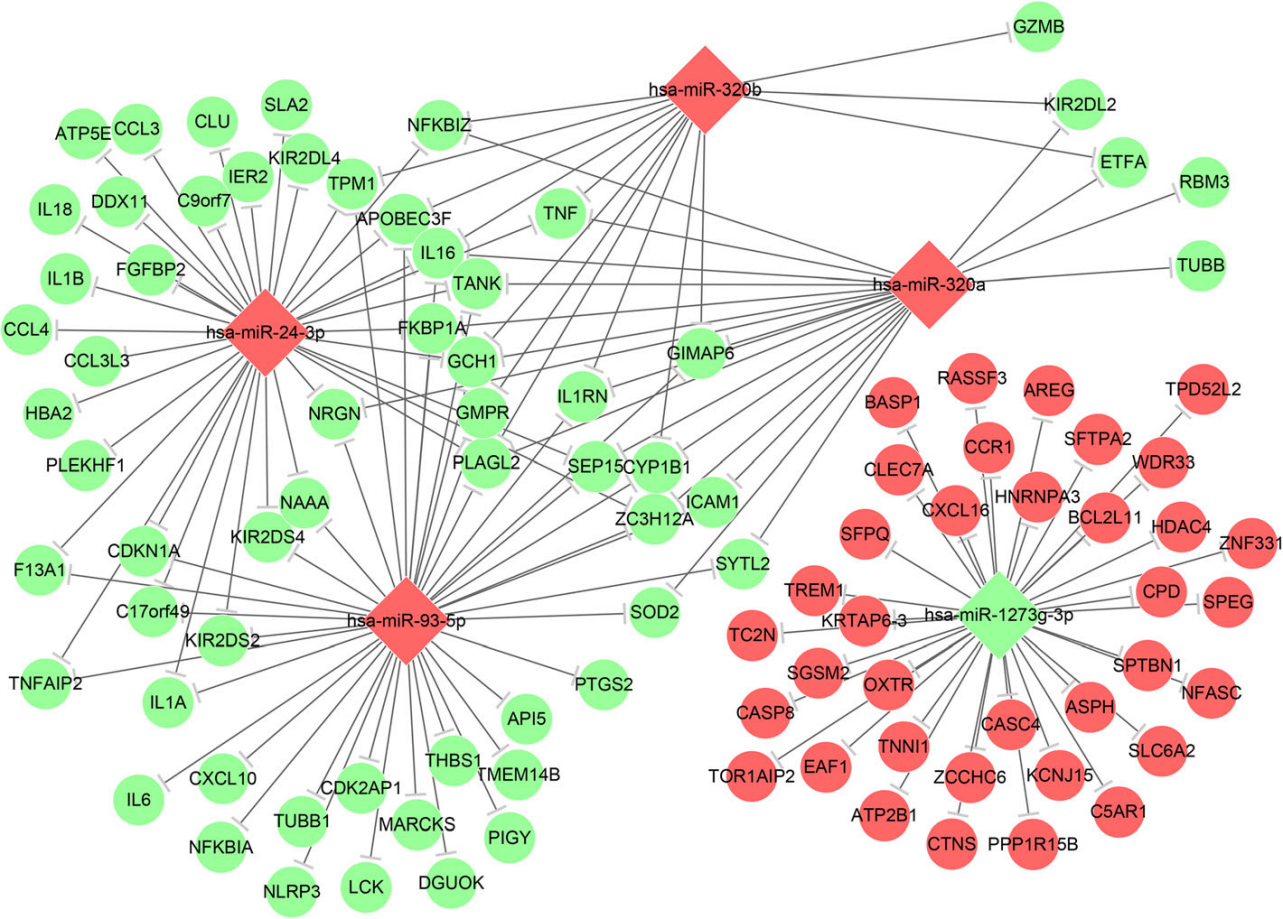
Regulation network between miRNAs and mRNAs. The negatively regulation of miRNA on dysregulated mRNAs was predicted and the regulation network was drawn by using Cytoscape software. Red and green color represents up- and down-regulated genes, respectively

**Fig. 6:**
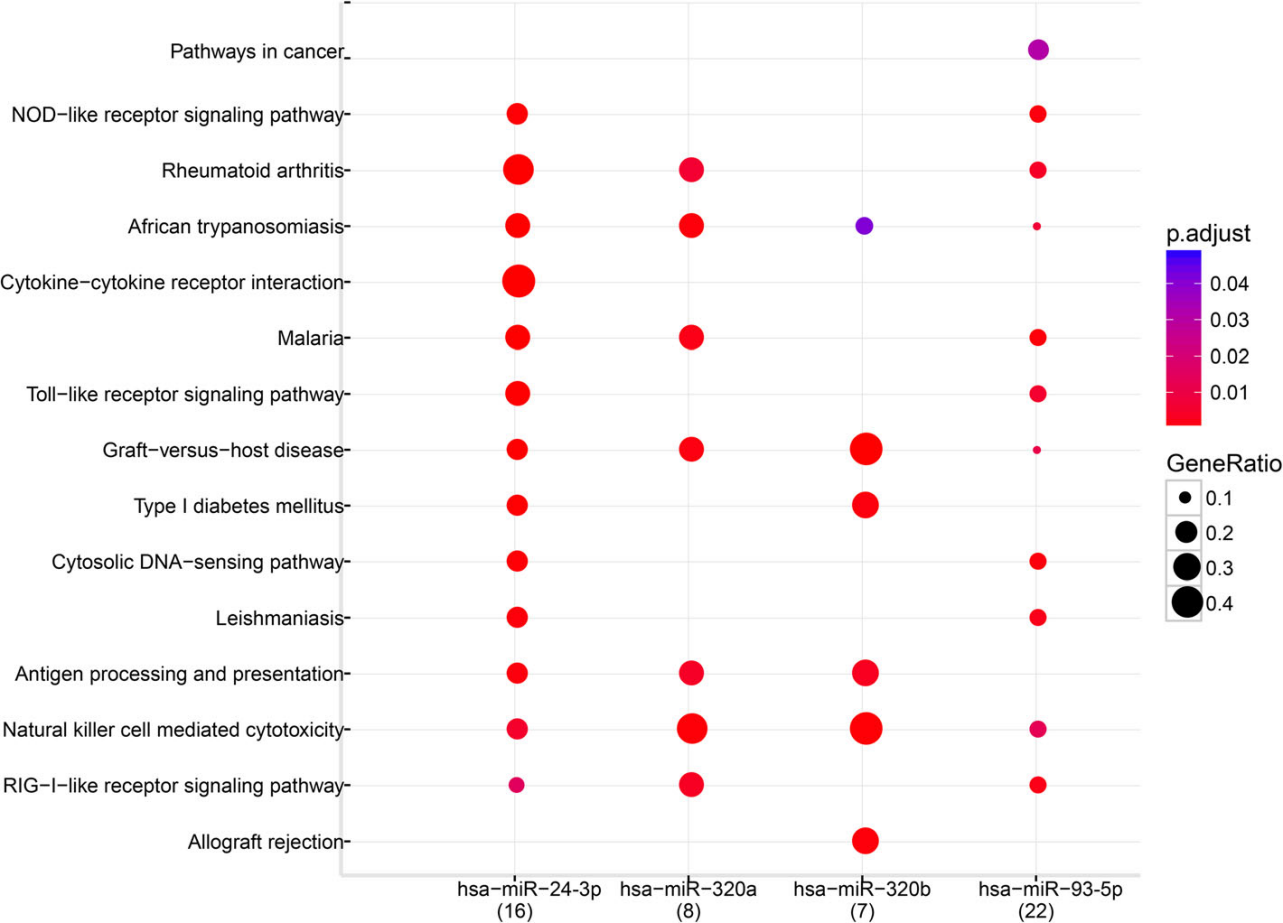
Pathway enrichment of dysregulated mRNAs regulated by miRNAs. The pathways in dysregulated mRNAs predicted by miRNAs were enriched by KEGG pathway enrichment analysis. “p adjust” represents the *P* value range. “Gene Ratio” represents the ratio of predicted target gene number in total gene number of each relevant pathway

**Fig. 7:**
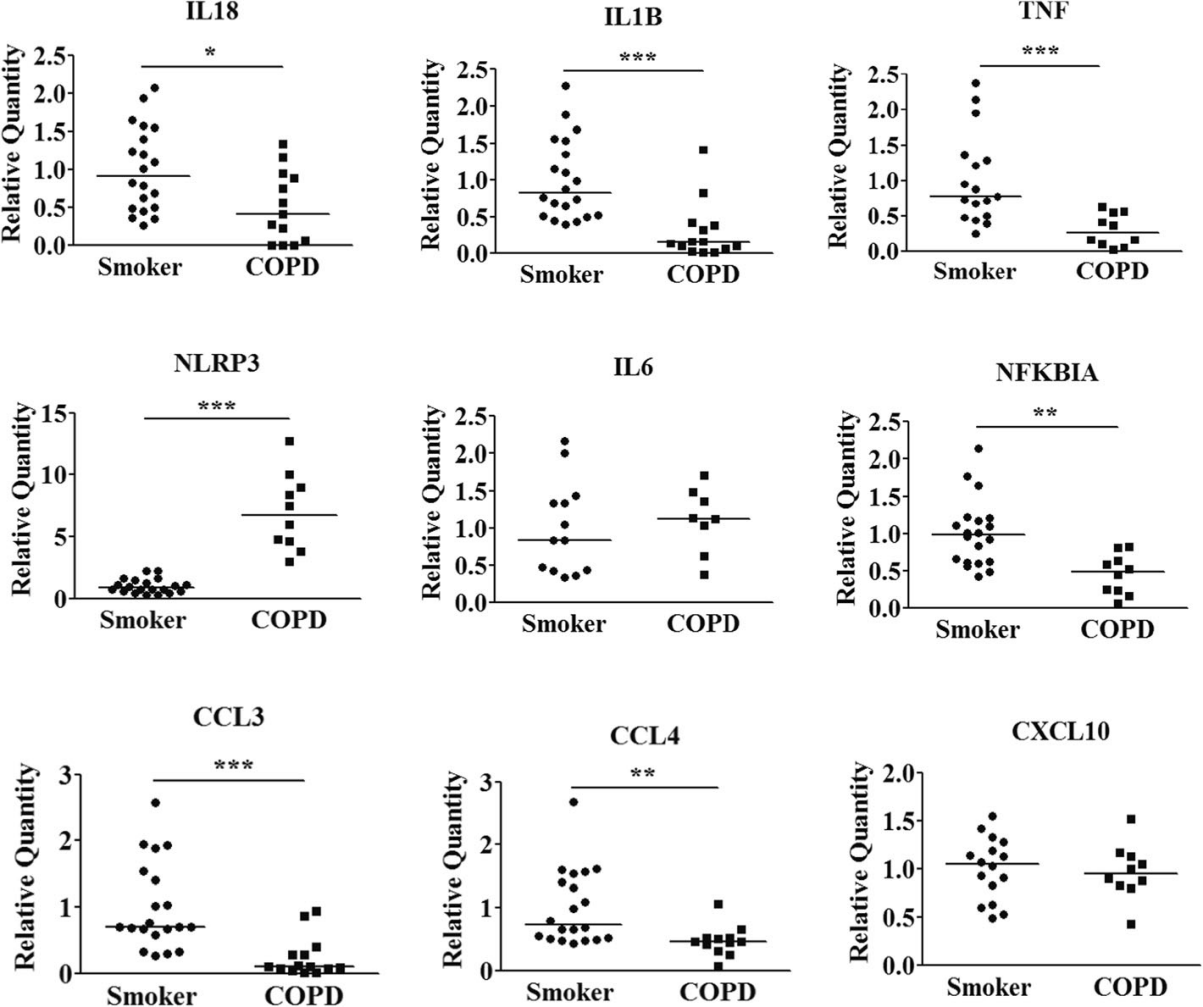
Validation of predicted target genes of miRNAs. The expression of predicted target genes of miRNAs in PBMCs of smokers and COPD patients was examined by qRT-PCR. Data are presented as 2^(−σσCt)^ relative to β-actin. ^*^ *P* < 0.05, ^**^ *P* < 0.01, ^***^ *P* < 0.001 compared with smokers by Mann Whitney *U* test.

Based on the genes negatively correlated with miRNAs from microarray, the predicted regulatory network between the dysregulated miRNAs (that were validated) and mRNAs in COPD patients was then analyzed (Fig. 5, Table 5). The KEGG pathway enrichment analysis indicated that the NOD − like receptor (NLR) and Toll − like receptor (TLR) signaling pathway relevant to the pathogenesis of COPD were significantly enriched (Fig. 6). The involved genes include IL18/IL1B/TNF predicted to be regulated by miR-24-3p, and NLRP3/IL6/NFKBIA by miR-93-5p for NLR pathway. The genes CCL3/CCL4/IL1B/TNF were predicted to be regulated by miR-24-3p, and IL6/CXCL10/NFKBIA by miR-93-5p for TLR pathway. The expression level of relevant predicted target genes of individual subjects were further validated by qRT-PCR. As shown in Fig. 7, the expression of IL18, IL1B, TNF, NFKBIA, CCL3 and CCL4 was validated, but not for NLRP3, IL6 and CXLC10.

**Table 5:** The pathway enrichment of dysregulated mRNAs regulated by miRNAs

miRNA	Pathway	Genes	Gene Ratio	*P* Value
miR-24-3p	Rheumatoid arthritis	IL1A/CCL3/CCL3L3/IL18/IL1B/TNF	6/16	2.37E-06
	African trypanosomiasis	HBA2/IL18/IL1B/TNF	4/16	2.47E-05
	Cytokine-cytokine receptor interaction	IL1A/CCL3/CCL4/CCL3L3/IL18/IL1B/TNF	7/16	2.53E-05
	Malaria	HBA2/IL18/IL1B/TNF	4/16	5.74E-05
	Toll-like receptor signaling pathway	CCL3/CCL4/IL1B/TNF	4/16	0.000645
	Chagas disease (American trypanosomiasis)	CCL3/CCL3L3/IL1B/TNF	4/16	0.000645
	Graft-versus-host disease	IL1A/IL1B/TNF	3/16	0.000741
	Type I diabetes mellitus	IL1A/IL1B/TNF	3/16	0.000743
	Cytosolic DNA-sensing pathway	CCL4/IL18/IL1B	3/16	0.001268
	NOD-like receptor signaling pathway	IL18/IL1B/TNF	3/16	0.001331
miR-320a	Natural killer cell mediated cytotoxicity	ICAM1/KIR2DL2/TNF	3/8	0.018267
	African trypanosomiasis	ICAM1/TNF	2/8	0.018267
	Graft-versus-host disease	KIR2DL2/TNF	2/8	0.018382
	Malaria	ICAM1/TNF	2/8	0.01936
	RIG-I-like receptor signaling pathway	TANK/TNF	2/8	0.029778
	Antigen processing and presentation	KIR2DL2/TNF	2/8	0.029845
	Rheumatoid arthritis	ICAM1/TNF	2/8	0.035321
	Folate biosynthesis	GCH1	1/8	0.072257
	Asthma	TNF	1/8	0.150454
	Graft-versus-host disease	GZMB/KIR2DL2/TNF	3/7	0.000379
miR-320b	Graft-versus-host disease	GZMB/KIR2DL2/TNF	3/7	0.000379
	Natural killer cell mediated cytotoxicity	GZMB/KIR2DL2/TNF	3/7	0.005999
	Allograft rejection	GZMB/TNF	2/7	0.008916
	Type I diabetes mellitus	GZMB/TNF	2/7	0.008916
	Antigen processing and presentation	KIR2DL2/TNF	2/7	0.019996
	Hypertrophic cardiomyopathy (HCM)	TNF/TPM1	2/7	0.019996
	Dilated cardiomyopathy	TNF/TPM1	2/7	0.020091
	Folate biosynthesis	GCH1	1/7	0.049597
	Asthma	TNF	1/7	0.117955
	African trypanosomiasis	TNF	1/7	0.117955
miR-93-5p	Malaria	IL6/ICAM1/THBS1	3/22	0.020688
	Cytosolic DNA-sensing pathway	IL6/CXCL10/NFKBIA	3/22	0.020688
	NOD-like receptor signaling pathway	NLRP3/IL6/NFKBIA	3/22	0.020688
	RIG-I-like receptor signaling pathway	CXCL10/NFKBIA/TANK	3/22	0.022958
	Leishmaniasis	IL1A/NFKBIA/PTGS2	3/22	0.022958
	Rheumatoid arthritis	IL6/ICAM1/IL1A	3/22	0.036896
	Toll-like receptor signaling pathway	IL6/CXCL10/NFKBIA	3/22	0.041751
	African trypanosomiasis	IL6/ICAM1	2/22	0.041751
	Prion diseases	IL6/IL1A	2/22	0.041751
	Bladder cancer	CDKN1A/THBS1	2/22	0.045977
	Malaria	IL6/ICAM1/THBS1	3/22	0.020688

## Discussion

MicroRNAs play important regulatory roles in cell differentiation, cell cycle and apoptosis. Due to the role of multiple gene regulation, miRNAs have received much attention as biomarkers and target for novel therapeutics. In COPD therefore, the role of miRNAs in disease pathogenesis is an attractive area of research. For the first time, we conducted a comprehensive analysis of both miRNA and mRNA expression in PBMCs from subjects with COPD and compared their expression profiles to smokers without airflow limitation. We identified 137 differentially-expressed miRNAs in PBMCs from COPD subjects compared with smokers without COPD. Among the selected 11 miRNAs, the dysregulated expression of 5 miRNAs including miR-24-3p, miR-93-5p, miR-320a, miR-320b and miR-1273g-3p were validated by qPCR.

Of the miRNA investigated in this study, miR-24-3p is of considerable interest. It has been reported that miR-24-3p was consistently upregulated during terminal differentiation of hematopoietic cells into a variety of lineages as well as during muscle and neuronal cell differentiation [20, 21]. MiR-24-3p might also function in cell proliferation [22, 23]. Upregulation of miR-24 is associated with a decreased DNA damage response upon etoposide treatment in highly differentiated CD8^+^ T cells [24], and miR-24 is a negative regulator of classical macrophage activation by LPS [25]. In this study, we found increased expression of miR-24-3p mainly in the T lymphocytes and monocytes, which might in part contribute to the increased number of CD8^+^ T cells in the lung [26] and to the impairment of host defenses in the lower respiratory tract due to the smoke related changes in the phenotype of alveolar macrophages of COPD patients [27].

As for the rest of dysregulated miRNAs reported in this study, we did not find the relevant evidences involving the pathogenesis of COPD, which implies the discrepancy of the miRNA profile between PBMCs and lung tissues. These miRNAs were mainly found dysregulated in cancers and other disorders like autoimmune diseases. For example, miR-93-5p was identified as a potential biomarker of various types of cancer such as acute myeloid leukemia [28] and laryngeal squamous cell carcinoma [29]. This could be due to the link between miR-93 and promotion of tumor growth, angiogenesis and metastasis [30, 31]. miR-93 is up-regulated in PBMCs from adult T-cell leukemia patients, and suppresses the expression of a tumor suppressor protein, tumor protein 53-induced nuclear protein 1 (TP53INP1) [32]. Four different transcripts have been reported in the database for miR-320 (miR-320a, b, c, and d) [33]. The seed region considered crucial for target binding remains the same for miR-320a, b, c, and d. The plasma level of miR-320a was found increased in patients with systemic lupus erythematosus (SLE) [34], and miR-320-3p is increased in the plasma of non-small cell lung cancer (NSCLC) patients [35]. The expression of miR-1273g-3p was found remarkably changed in Human Umbilical Vein Endothelial Cells (HUVECs) under acute glucose fluctuations, which was demonstrated to contribute to endothelial dysfunction and autophagy [36]. MiR-1273 expression is also increased in the pancreas of mouse model of pancreatic cancer [37].

By analyzing the regulation network between the dysregulated miRNA and mRNA, we predicted the negative regulatory role of miRNAs on a total of 36 over expressed and 61 under expressed mRNAs. The KEGG pathway enrichment analysis indicated that the NOD − like receptor (NLR) signaling pathway and Toll − like receptor (TLR) signaling pathway are the top 2 pathways likely involved in the pathogenesis of COPD, with mir-24-3p and miR-93-5p being predicted to regulate the relevant genes in both pathways. As down-stream factors of the NLR and TLR pathway, the mRNA levels of pro-inflammatory mediators including IL-18, IL-1β, CCL3, CCL4, and TNF were found down-regulated in PBMCs of COPD patients in this study. To the best of our knowledge, the down-regulation of these mRNAs in PBMCs of COPD patients has not been previously reported. In fact, a previous study performed on human peripheral lung tissue obtained from non-smokers, smokers and COPD patients revealed the similar trends of expression level of pro-inflammatory mediators, where the level of IL-8, IL-6, IL-1β and TNF-α showed the decreased trend in COPD patients compared with smokers [38]. Instead, most previous studies showed elevated expression levels of these mediators in serum or lung [39]. We suspect that certain changes in the cytokine expression profile may happen when the peripheral immune cells infiltrate the local inflammatory sites in the lung. The reduced expression of TLR2 has been found in the alveolar macrophages of smokers and COPD patients, which was associated with the impairment of host defenses in the lower respiratory tract [27]. Furthermore, decreased cytokine and chemokine mRNA expression in bronchoalveolar lavage cells from asymptomatic smokers has been reported [40].

In addition, NFKBIA predicted to be regulated by miR-93-5p- was the other down-regulated gene involved in enriched pathways. In unstimulated cells, NF-κB is found in the cytoplasm in an inactive non-DNA binding form, associated with its inhibitory protein κBα (IκBα, coded by NFKBIA gene), IκBα degradation unmasks the nuclear localization signal present in NF-κB, allowing it to enter the nucleus, bind DNA, and initiate gene transcription [41]. In the present study, the down-regulation of IκBα supposedly trigger the activation of NF-κB, whose expression was also higher in PBMCs of COPD patients. The coexistence of decreased level of IκBα and pro-inflammatory mediators in COPD patients was also reported on lung tissue [38]. Besides trigging the expression of pro-inflammatory mediators, NF-κB activation may also be related with the disordered apoptosis of T-cell hybridoma cell line [42]. Thus, the decreased expression of IκBα may contribute to the dysregulated apoptosis of T cells in COPD [43].

Overall, through miRNAs and mRNAs expression profiling in smokers and COPD patients, we identified the dysregulated miRNAs and mRNAs in PBMCs from COPD patients. We further analyzed the regulation network between miRNA and mRNA, where NLR and TLR was the most enriched pathways. Among them the regulation of IL-18, IL-1β, TNF, CCL3 and CCL4 by miR-24-3p, and IκBα by miR-93-5p may provide the clue for potential investigations.

## Conclusions

The expression of miRNA and mRNA were dysregulated in PBMCs of COPD patients compared with smokers without airflow limitation. The regulation network between the dysregulated miRNA and mRNA may provide potential therapeutic targets for COPD.

## Abbreviations

COPD: Chronic obstructive pulmonary disease
NLR: NOD − like receptor
PBMC: Peripheral blood mononuclear cell
TLR: Toll − like receptor
